# Puzzle Pieces: Neural Structure and Function in Prader-Willi Syndrome

**DOI:** 10.3390/diseases3040382

**Published:** 2015-12-17

**Authors:** Katherine E. Manning, Anthony J. Holland

**Affiliations:** 1 Department of Psychiatry, University of Cambridge, Cambridge, CB2 8AH, UK; E-Mail: ajh1008@medschl.cam.ac.uk; 2 National Institute for Health Research (NIHR) Collaborations for Leadership in Applied Health Research and Care (CLAHRC) East of England, Cambridge, CB2 8AH, UK; 3 Cambridgeshire and Peterborough Mental Health Services (NHS) Foundation Trust, Cambridge, CB21 5EF, UK

**Keywords:** Prader-Willi syndrome, brain, systematic review, neuroimaging, post-mortem

## Abstract

Prader-Willi syndrome (PWS) is a neurodevelopmental disorder of genomic imprinting, presenting with a behavioural phenotype encompassing hyperphagia, intellectual disability, social and behavioural difficulties, and propensity to psychiatric illness. Research has tended to focus on the cognitive and behavioural investigation of these features, and, with the exception of eating behaviour, the neural physiology is currently less well understood. A systematic review was undertaken to explore findings relating to neural structure and function in PWS, using search terms designed to encompass all published articles concerning both *in vivo* and post-mortem studies of neural structure and function in PWS. This supported the general paucity of research in this area, with many articles reporting case studies and qualitative descriptions or focusing solely on the overeating behaviour, although a number of systematic investigations were also identified. Research to date implicates a combination of subcortical and higher order structures in PWS, including those involved in processing reward, motivation, affect and higher order cognitive functions, with both anatomical and functional investigations indicating abnormalities. It appears likely that PWS involves aberrant activity across distributed neural networks. The characterisation of neural structure and function warrants both replication and further systematic study.

## 1. Introduction

Prader-Willi Syndrome (PWS) is a neurodevelopmental disorder of genomic imprinting resulting in the lack of paternal expression of maternally-imprinted genes at chromosomal locus 15q11-13, most commonly caused by a deletion of this region on the paternal chromosome (*ca*. 70% cases), maternal uniparental disomy (UPD; *ca*. 25% cases), or a defect in the regulating imprinting centre (*ca*. 3%–5% cases). Affecting around one in 25,000–29,000 live births, PWS presents with a characteristic, although variable, phenotype [1,2]. Most prominently, infants are born with severe hyptonia and show an initial failure to thrive, which is replaced during preschool years by an insatiable appetite, necessitating external restriction of food and commonly resulting in life-threatening obesity if such restrictions are not in place. Relative growth and sex hormone deficiencies mean that short stature is common and sexual development disrupted, and a typical facial phenotype is often present. Beyond these obvious effects, such hormonal deficiencies can also be expected to influence neural development. The role of growth hormone in the brain is not fully understood, but receptors are found throughout the brain and research has suggested potential effects of growth hormone supplementation in PWS on behaviour and cognition [3]. Similarly, although not the sole influence of sex on neural structure and function, sex hormones play an important role in sexual differentiation during brain development [4]. The treatment of these deficiencies, as has become common in PWS, may again alter the course of brain development.

The characteristics described thus far strongly suggest a disorder of hypothalamic control. However, in addition to these core features, PWS is typically associated with mild to moderate intellectual disability, a high pain threshold and a variable range of social and behavioural difficulties, including temper outbursts, need for routine, skin picking, repetitive and ritualistic behaviours, and poor social functioning, as well as a propensity to psychiatric disturbance [5,6,7]. Thus, the effects of the genetic aberration resulting in PWS are diverse and are likely to involve a widespread atypical pattern of development of the brain. While the characterisation of PWS at the cognitive and behavioural levels has received considerable attention, with the exception of the eating behaviour, much less is known about the neural endophenotype. Such behaviours are also likely to be shaped and maintained by environmental factors, however, a greater elucidation of the neural underpinnings of these behaviours would aid our understanding of the likely complex biopsychosocial interactions that result in such behaviours and why they persist over time. Such knowledge will lead ultimately to the development of treatments, pharmacological or psychological, which could have a significant effect on quality of life for individuals with PWS and those who support them.

Increased understanding of the brain in PWS will also offer insight into the role of genomic imprinting in the brain and the purposes this may serve within the typically-developing population, with the paternal contribution disrupted in PWS, as well as gene dosage effects of the maternal contribution in UPD. Angelman’s syndrome (AS) is the sister imprinting disorder of PWS, arising from the lack of maternal expression of paternally-imprinted genes located at the same critical region on chromosome 15, and presents with a different phenotype including greater severity of intellectual disability, seizures, ataxia and speech impairment. Perhaps the most prominent evolutionary theory of genomic imprinting discussed in relation to PWS is kinship theory, which suggests that gender of origin imprinted genes that are only expressed from the paternal allele are particularly involved in growth and attainment of resources, including in placental functioning prenatally, whilst alleles of genes that are only expressed from the maternal line appear more integral to balancing the needs of the offspring with costs to the matrilineal relatives [8]. Better knowledge about the effects of aberrant genomic imprinting on the PWS neural endophenotype may help support or refute these claims.

## 2. Literature Search

**Figure 1:**
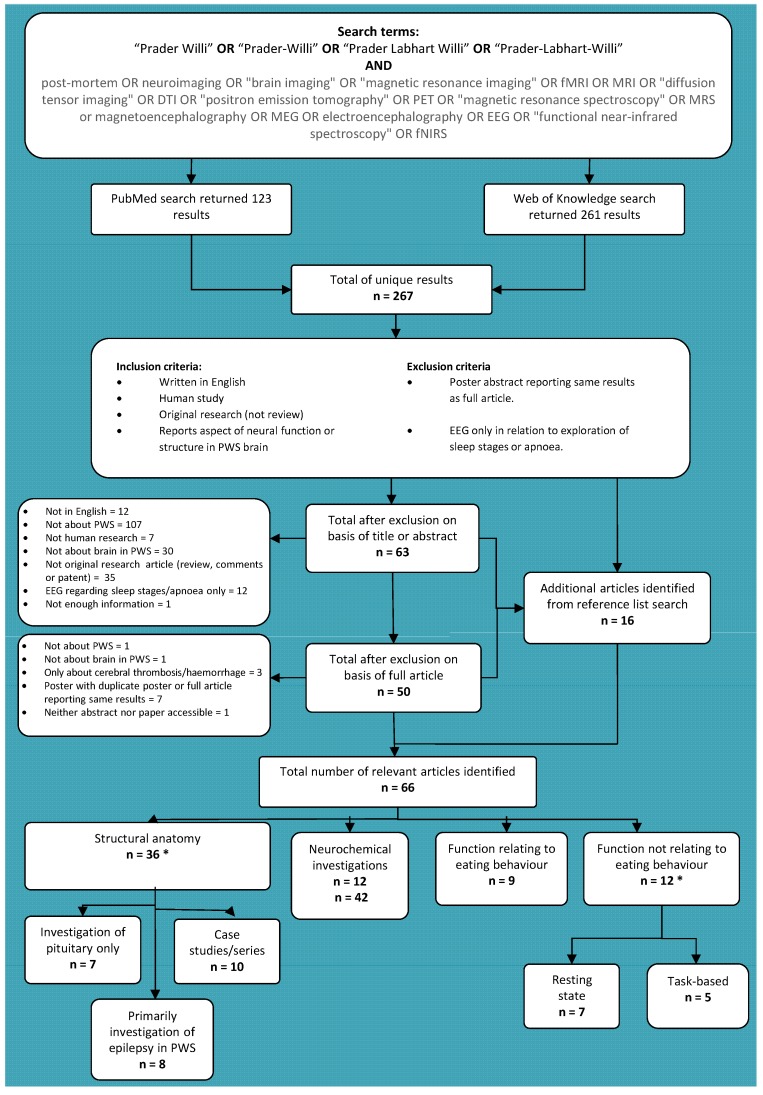
Article identification process for systematic literature review of neural structure and function in Prader-Willi syndrome (PWS). Literature search was carried out on 28 April 2015. Note that subcategories (e.g., case studies/series) within each main category (e.g., structural anatomy) are not mutually exclusive and references may be featured in more than one subcategory. *: One study features in both categories.

A systematic review was undertaken to explore findings relating to neural structure and function in PWS, using the search terms and inclusion pathway illustrated in Figure 1 below. The PubMed and Web of Knowledge databases were searched using a search term designed to identify all published articles (including conference abstracts) concerning both *in vivo* and post-mortem studies of neural structure and function in PWS. All articles written in English and reporting an original study of neural structure or function in PWS were included and review articles and reference lists were searched for additional relevant articles not already identified. Articles reporting electroencephalography (EEG) solely in relation to sleep stages or apnoea were excluded, since they tended to describe sleep stage duration only, as were articles solely reporting haemorrhage, stroke or cerebral thrombosis. Most of the latter reports were case studies and such insult to the brain was felt to be unlikely to be highly informative to an understanding of PWS itself, but more related to the effects of these other conditions. As such, they were beyond the scope of this review. An initial round of screening excluded articles according to title or abstract which clearly did not meet the inclusion criteria, before eligibility of remaining articles was assessed. This supported the general paucity of research in this area, returning only 66 relevant unique articles, including case studies and conference abstracts.

Relevant articles were subdivided according to whether they concerned neural structure at the macrostructural level, neurochemical investigations and neuroanatomy at the cellular level, neural function in relation to eating behaviour or neural function relating to other aspects of the PWS phenotype. This further highlighted that neural structure and function have typically been considered in isolation, with the majority of functional investigations concerning eating behaviour in PWS and over half of the structural studies reporting case studies or investigations of the pituitary gland alone. Further details of all articles, including brief summaries of methodologies, sample characteristics and key findings can be found in Table 1, Table 2 and Table 3.

## 3. Anatomical Structure of the PWS Brain

Given the many features of PWS indicative of hypothalamic dysfunction, a significant proportion of studies regarding the anatomy of the brain in PWS have focused on the hypothalamic-pituitary area. Findings have varied, and, while indicating possible abnormalities, neither evidence for hypothalamic pituitary anomaly nor the form of any defect is conclusive. Two case studies of children with PWS reported normal pituitary evaluations using magnetic resonance imaging (MRI) [9,10], while another found a reduced pituitary diameter but no further neuropathologies [11]. Retrospectively evaluating the MRIs of 91 children with PWS, Iughetti *et al.* (2008) reported decreased pituitary height in 45 and absence of posterior pituitary bright spot in six [12], while Tauber *et al.* (2000) also reported pituitary hypoplasia in 60% of a retrospective sample of 16 children with MRI available, regardless of whether or not they had received GHT [13]. Grugni *et al.* (2000) reported 10 of 17 PWS participants presenting with pituitary hypoplasia and three with empty sella, with only four showing a normal pituitary gland [14]. However, these studies did not directly compare individuals with PWS to control samples.

**Table 1:** Studies reporting macrostructural anatomy in PWS.

Author	Methods	Sample	Key findings
Cacciari *et al.* 1990 [9]	MRI—pituitary evaluation only	1 PWS patient in larger study of 101 patients (15 y)	No abnormality found.
Crino *et al.* 2008 [10]	MRI—pituitary evaluation only	8 y old male with PWS	No abnormality found.
Fan *et al.* 2005 [15]	Retrospective case reviews of available MRI data	MRI available for 9/10 patients with seizures from total PWS sample of 56 (31 males, 1–37 y)	MRI normal for 8/9; haemorrhage in 1/9.
Gilboa & Gross-Tsur 2013 [16]	Retrospective case reviews of available imaging data for patients: MRI, CT, ultrasonography	Imaging data available for 59 of 125 PWS patients (50% male, 1 month–48 y): MRI for 30; CT for 17; ultrasonography for 12	Abnormalities in 20/59, incl. 4/5 with epilepsy: including ventriculomegaly (3/59); thalamic abnormalities (3/59); partial corpus callosum agenesis (3/59); disturbed myelination (3/59); partial empty sellar (1/59).
Grugni *et al.* 2000 [14]	MRI—pituitary only.	17 PWS patients (9 males, mean 20 y)	Pituitary hypoplasia (10/17); empty sella (3/17).
Hashimoto *et al.* 1998 [17]	MRI and ^1^H-MRS	5 PWS patients (2 males, 1–14.5 y) 37 control infants and children (1–15 y)	Abnormalities in 5/5: mild ventriculomegaly (4/5); frontal cortical atrophy (3/5); small brainstem (1/5); delayed myelination (1/5). Metabolite abnormalities: Reduced NAA peak in; NAA:Choline and NAA:Creatine ratios tended to be lower in PWS group with a signification relationship between these metabolite rations and developmental level.
Hayashi *et al.* 1992 [18]	Post-mortem	6 month old female with PWS	No pituitary abnormality; abnormalities of cortical gyrification, cerebellar white matter and dentate gyrus.
Honea *et al.* 2012 [19]	MRI	23 participants with PWS (8 males, 10–39 y) 25 typically-developing control participants (11 males, 10–48 y)	Deletion *vs.* UPD: reduced grey matter volume in PFC & temporal cortex and reduced white matter volume in parietal cortex in deletion; reduced grey and white matter volume in OFC and limbic cortex in UPD.
Iughetti *et al.* 2008 [12]	Retrospective case reviews of available MRI data	91 children with PWS (49 males, 0.7–16.8 y)	Abnormalities in 61/91: reduced pituitary height (45/91); absence of posterior pituitary bright spot (6/91); ventriculomegaly (8/91); thin corpus callosum (2/91).
Kumada *et al.* 2005 [20]	Retrospective case reviews of available MRI data	Imaging data available for 3/4 patients with PWS and epilepsy (1 males, 3–12 y) in larger study of chromosomal abnormalities and epilepsy	Cerebral atrophy in 1/3.
Leonard *et al.* 1993 [21]	MRI	4 children with PWS (3 males, 3–10 y) 6 with AS (3 males, 2–7 y)	Abnormality in sylvian fissure of 1/4 PWS children (unilateral), but 6/6 AS (2 bilateral, 4 unilateral).
Linnemann *et al.* 1999 [11]	MRI case study	6 y old male with PWS	Small flat pituitary; no further abnormalities.
Lukoshe *et al.* 2013 [22]	MRI	20 children with PWS (9 males, 6–17 y) 11 sibling control participants (8 males, 7–15 y)	Significantly reduced brainstem volume in PWS group compared to control group; trend to reduced white matter volume and total cortical surface area in PWS. Greater ventriculomegaly and surface CSF in UPD compared to deletion subtypes.
Lukoshe *et al.* 2014 [23]	MRI	24 children with PWS (9 males, 6–18 y) 11 sibling control participants (8 males, 7–15 y)	Decreased cortical complexity in four clusters in frontal, temporal and parietal lobes. Cortical complexity positively correlated with IQ. Greater cortical thickness and lower cortical complexity in some clusters in UPD.
Mantoulan *et al.* 2011 [24]	MRI	9 PWS teenagers (6 males, 12.7–18.6y, mean 16.4 y) 9 typically developing young adults (6 males, mean 21.2 y)	No abnormalities found.
Maski *et al.* 2009 [25]	Retrospective case reviews of available “neuroimaging” data: unspecified modality	Imaging data available for 14/21 PWS patients with seizures	Unspecified non-focal abnormalities in 3/14.
Miller *et al.* 1996 [26]	MRI	15 individuals with PWS (6 males, 2–28 y) 16 age- & sex-matched group with isolated growth hormone deficiency (10 males, 3–14 y) 49 age-matched typically-developing controls from previous literature (1–29 y)	No significant difference in pituitary height. Posterior pituitary bright spot absent in 3/15 PWS participants and reduced in 1/15.
Miller *et al.* 2006 [27]	MRI	17 PWS participants (11 males, 4–39 y) 18 individuals with early-onset morbid obesity (EMO) (9 males, 4–22 y) 21 typically-developing siblings (8 males, 3.5–43 y)	White matter abnormalities in 6/8 adults with PWS and 5 participants with EMO, but 0/9 children with PWS and none in typically-developing controls participants.
Miller *et al.* 2007 [28]	MRI	27 PWS participants (16 males, 3 m–39 y) 13 EMO participants (4 males, 4–16 y) 26 typically developing siblings (10 males, 2 m–43 y).	Incomplete insular closure in PWS, compared to both sibling and EMO control groups.
Miller *et al.* 2007 [29]	MRI	20 PWS participants (12 males, 3 m–39 y) 16 individuals with EMO (8 males, 15 m–22 y) 21 typically-developing siblings (10 males, 2–43 y)	Abnormalities in all 20 PWS participants: ventriculomegaly (20/20); incomplete closure of the insula (13/20); sylvian fissure polymicrogyria (12/20); decreased parietal-occipital volume (10/20). None of these abnormalities in either EMO or sibling control groups.
Miller *et al.* 2008 [30]	MRI—pituitary evaluation only	27 individuals with PWS (16 males, 3 m–39 y) 16 individuals with EMO (6 males, 4-22 y) 25 typically-developing siblings (10 males, 2–43 y)	Pituitary abnormalities in 20/27 PWS, 10/16 EMO & 2/25 controls. Abnormality not specific to PWS.
Miller *et al.* 2009 [31]	MRI	16 PWS participants (10 males, mean 16.53 y) 12 EMO participants (4 males, mean 9.25 y) 15 typically-developing siblings (5 males, mean 12.08 y)	Reduced cerebellar volume and cerebellar/cerebral ratio in PWS and EMO compared to sibling group, but no difference between PWS and EMO.
Ogura *et al.* 2011 [32]	MRI	12 adults with PWS (6 males, 19–31 y) 13 age- & sex-matched controls (6 males, 19–29 y)	Reduced total brain, grey matter and white matter volume, and focally reduced OFC and somatomotor area volume in PWS.
Stevenson *et al.* 2004 [33]	MRI and post-mortem	Post-mortem: 5 month, 9 month, & 3.5 y old with PWS (2 males) MRI: 9 month old female with PWS	Cortical grey & white matter abnormalities in 3/4; midbrain, hindbrain & cerebellar abnormalities in 1/4; normal in 1/4.
Takeshita *et al.* 2013 [34]	Retrospective case reviews of available imaging data for patients: MRI, CT	MRI available for 9/31 PWS patients with seizures (6 males, neonatal–3 y)	Diffuse atrophy in 1/9, but appears to have followed some injury to the brain of undisclosed nature. 8/9 within normal limits.
Tauber *et al.* 2000 [13]	Retrospective case reviews of available MRI data—pituitary only	MRI for 16/28 with PWS (mean 11.8 y)	Pituitary hypoplasia in 10/16.
van Nieuwpoort *et al.* 2011 [35]	MRI—pituitary only	15 adults with PWS (4 males, 19.2–42.9 y) 14 typically-developing siblings (7 males; 17.5–41.3 y)	Reduced anterior pituitary size in 12/15 adults with PWS compared to sibling group.
Vendrame *et al.* 2010 [36]	Retrospective case reviews of available MRI data	Imaging available for 20/30 PWS patients with seizures (6 males, 4–21 y)	Abnormalities in 5/20: ventriculomegaly (4/20); diffuse cortical atrophy (1/20).
Verrotti *et al.* 2015 [37]	Retrospective case reviews of available imaging data for patients: MRI, CT	Imaging data available for 35/28 PWS patients with seizures (22 males, seizure onset 2 days–11 y, age at imaging evaluation unspecified): MRI for 25; CT for 10	Abnormalities in 11/35: ventriculomegaly (5/35), cortical atrophy (4/35), corpus callosum hypoplasia (1/35), periventricular leukomalacia (1/35).
Yamada *et al.* 2006 [38]	DTI	8 participants with PWS (6 males, 8–29 y) 8 age- & sex-matched typically-developing controls (6 males, 8–29 y)	Atypical diffusivity indicating abnormalities of frontal white matter, posterior limb of internal capsule, and splenium of corpus callosum.
Yoshii *et al.* 2002 [39]	MRI case study	40 week old female with PWS	Abnormal gyrification and cortical grey-white matter boundaries; partially uncovered right insula.

AS: Angelman’s syndrome; BMI: body mass index; CSF: cerebrospinal fluid; CT: computerised tomography; DTI: diffusion tnsor imaging; EMO: early-onset morbid obesity; ^1^H-MRS: proton magnetic resonance spectroscopy; MRI: magnetic resonance imaging; IQ: intelligence quotient; m: months; NAA: *N*-acetylaspartate; OFC: orbitofrontal cortex; PFC: prefrontal cortex; PWS: Prader-Willi syndrome; UPD: uniparental disomy; y: years.

**Table 2:** Studies reporting neural function in PWS at the macrostructural level.

Study	Methods	Sample	Mai findings
Akefeldt *et al.* 1997 [40]	EEG: Auditory brainstem response.	7 participants with PWS (6 males, 4–25 y) 7 control participants with intellectual disability (5 males, 5–26 y)	Atypicalities of auditory brainstem response compared to control group and laboratory reference values.
Dimitropoulos & Schultz 2008 [41]	fMRI: response to high *vs.* low calorie food images.	9 participants with PWS (3 males, 8–38 y) 10 IQ and BMI matched control participants (4 males, 19–29 y)	Increased amygdala, hypothalamus, insula and OFC activity when fasted in response to images of high *vs.* low calorie foods.
Halit *et al.* 2008 [42]	EEG: face and gaze perception.	8 adults with deletion subtype of PWS (20–53 y) 8 with UPD subtype of PWS (19–52 y)	Behavioural performance impaired for both genotypes, but ERP showed increased impairment in the deletion group: N170 amplitude larger for averted *vs.* direct gaze and inverted *vs.* upright faces in UPD, but not deletion group.
Hinton *et al.* 2006 [43]	PET: pre- and post-meal (400 & 1200 kcal) response to food images.	13 participants with PWS (22–42 y)	Increased activity in medial OFC, temporal cortex and PFC usually found not seen in PWS following meals when viewing food images.
Hinton *et al.* 2006 [44]	PET: response to high and low incentive food images.	13 participants with PWS (22–42 y)	Increased activation of amygdala and medial OFC typically seen in response to high *vs.* low incentive foods not found.
Holsen *et al.* 2006 [45]	fMRI: pre- and post-meal (500 kcal) response to food images and control animal images	9 participants with PWS (1 male, mean 14.7 y) 9 age matched typically-developing controls (3 males, mean 14.4 y)	PWS group showed reduced activity when viewing food *vs.* animal images in medial PFC and OFC in pre-meal condition than control group and greater medial PFC, insula, parahippocampal gyrus and amygdala activity in post-meal condition.
Holsen *et al.* 2009 [46]	fMRI: pre- and post-meal (500 kcal) responses to food images and control animal images.	9 participants with PWS deletion subtype (2 males; mean 24.4 y) 9 participants with PWS UPD subtype (3 males; mean 20.8 y) 9 healthy weight controls (3 males; mean 23.6 y)	Both PWS subtypes showed atypical response in both pre- and post-meal conditions compared to control group. Deletion *vs.* UPD: greater activity in deletion group in frontal/limbic areas, especially medial PFC and amygdala, in both pre- and post-meal conditions; greater activity in dorsolateral PFC and parahippocampal gyrus in UPD group in post-meal condition only.
Holsen *et al.* 2012 [47]	fMRI: pre- and post-meal 500 kcal) responses to food images and control animal images.	14 participants with PWS (2 males, mean 23.3 y) 14 BMI & age-matched control participants (5 males; mean 25.0 y) 15 healthy weight age-matched controls (6 males; mean 23.1 y)	Pre-meal: increased activity when viewing food *vs.* non-food images in nucleus accumbens and amygdala in PWS than either control group control group, and lower in hypothalamus and hippocampus. Post-meal: increased activity in hypothalamus, amygdala and hippocampus in PWS than either control group, but higher dorsolateral PFC and OFC in obese group not seen in PWS or healthy weight control group.
Key & Dykens 2008 [48]	EEG: N1 & P3 ERP response to food images according to categorisation/discrimination of food composition and quality.	9 participants with deletion subtype of PWS (2 males, mean 22.9 y) 8 participants with UPD subtype of PWS (3 males, mean 22.4 y) 9 age-matched control participants (4 males, mean 21.7 y)	N1 ERP suggested deletion group early processing by categorising mainly according to quantity, whilst UPD did so by quality and suitability for consumption more similarly to the control group. Later P3 response showed deletion group could discriminate foods by combinational suitability, but this later processing response was greater than that seen in UPD. Later P3 motivational processing of food images not seen in control group.
Kim *et al.* 2006 [49]	Resting-state PET while fasted.	16 children with PWS (9 males, mean 4.2 y) 7 typically-developing siblings/relatives (4 males, mean 4.0 y)	PWS group showed decreased metabolism in right superior temporal gyrus and left verebellar vermis, and increased metabolism in right OFC, bilateral medial PFC, right inferior and left superior frontal cortex, bilateral ACC, right temporal pole and left uncus.
Klabunde *et al.* 2015 [50]	fMRI: sessions where participants engaged in skin picking compared to sessions where they did no.	17 participants with PWS (11 males, mean 15.7 y)	2 main clusters showing greater activation during skin picking: right ACC & right middle frontal gyrus; primary somatosensory cortex, left inferior parietal lobule, supplementary motor area, left middle frontal gyrus and right posterior insula. Self-injury trauma scale scores negatively correlated with right insula and left precentral gyrus activity.
Mantoulan *et al.* 2011 [24]	Resting-state PET	9 PWS teenagers (6 males; 12.7–18.6y, mean 16.4 y) 9 typically developing young adults (6 males; mean 21.2 y)	Hypoperfusion in PWS, most strongly in ACC and superior temporal regions, but also in right orbitofrontal gyrus and postcentral gyrus. Positive correlations between Child Behaviour Checklist (CBCL) scale scores and rCBF in ACC (activity, social, & attention scales), superior temporal gyrus (attention & social scales), and superior frontal gyrus (activity scale). Negative correlation between rCBF in ACC and CBCL depression scale score.
Miller *et al.* 2007 [51]	fMRI: response to food images following glucose load.	8 participants with PWS (6 males, mean 25 y) 8 typically-developing siblings (4 males, mean 27 y)	Significantly increased activity ventromedial PFC in PWS group when viewing food images following glucose load.
Ogura *et al.* 2013 [52]	Resting state PET	12 participants with PWS (6 males, 19–31 y) 13 age- & gender-matched controls (6 males, 19–29 y)	Decrease metabolism in PWs group in the lingual gyri, cerebellum, right thalamus and left insula. Increased metabolism in bilateral angular and inferior frontal gyri and left middle frontal gyrus. Negative correlation between questionnaire score of eating severity and rCBF in left insula.
Pujol *et al.* 2014 [53]	Resting state fMRI	24 adults with PWS (12 males, mean 26.3 y) 20 adults with Down’s syndrome (10 males, mean 24.5 y) 20 adults with William’s syndrome (11 males, mean 25.2 y) 80 young adults (45 males, mean 26.4 y) 71 children (30 males, mean 9.6 y) 53 older adults (24 males, mean 67.4 y)	Young adults showed sensorimotor system activity positively correlating with motion, suggesting system specific effects. Seen to some extent in children and older adults but also wider effects of motion. Greater motion in genetic disorders, and specific motion-connectivity correlations: PWS group shoed mostly frontal and temporal lobe correlations with motion, but also significant in the dorsal ACC; in Down’s syndrome group was with anterior and dorsal regions; and in William’s syndrome were diffuse correlations across grey matter voxels.
Shapira *et al.* 2005 [54]	fMRI: before and after glucose load.	3 participants with PWS (1 male, 25–38 y)	Delay in activation of brain areas associated in satiety response in previous study of participants without PWS, including hypothalamus, insula, ventromedial PFC and nucleus accumbens following oral glucose load.
Stauder *et al.* 2002 [55]	EEG: P3 ERP response to visual and auditory oddball tasks.	10 adults with PWS (5 males, mean 30.8 y) 10 typically-developing controls (3 males, mean 23.2 y)	Markedly decreased P3 response in both visual and auditory tasks in PWS compared to control group, and most strongly for the auditory task.
Stauder *et al.* 2005 [56]	EEG: N200 and P300 ERPs during response inhibition on Go-Nogo task.	11 participants with deletion subtype of PWS (7 males, mean 26.7 y) 11 participants with UPD subtype of PWS (4 males, mean 27.7 y) 11 typically-developing control participants (6 males, mean 27.3 y)	Behavioural task performance poorer for both PWS groups compared to control group. N200 amplitude didn’t show normal peak in either PWS group, suggesting impaired early modality specific inhibition in both UPD and deletion, but only UPD group showed impaired P300 modulation, indicative of later general inhibition.
Woodcock *et al.* 2010 [57]	fMRI: set-shifting task.	8 participants with PWS (5 males, mean 20.7 y) 8 age & gender matched typically-developing control participants (mean 21 y)	Control group showed increased activity compared to the PWS group in frontoparietal regions during switching, including in ventromedial PFC and posterior parietal cortex. PWS tended to show ventromedial PFC deactivation instead. Group interactions: Greater activity in hippocampus, amygdala, thalamus and putamen during switching in control group than PWS group, driven by deactivation of these areas by PWS group during switching alongside activation in the control group. The reverse pattern was found for the anterior poles.
Zhang *et al.* 2013 [58]	Resting state fMRI	21 participants with PWS (11 males, mean 7.3 y) 18 sibling control participants (8 males, mean 11.1 y)	ALFF greater in PWS than control group in ventrolateral PFC, ACC, inferior parietal lobe and left insula, and decreased in the medial and dorsolateral PFC, hippocampus, pre- and post-central gyri, and left OFC. DMN: reduced functional connectivity pairwise between medial PFC, inferior parietal lobe and precuneus, and between precuneus and inferior parietal lobe. PFC network: reduced functional connectivity between dorsolateral PFC and OFC, and increased functional connectivity between ventrolateral PFC and both OFC and dorsolateral PFC. Core network: increased ACC-insula functional connectivity. Motor sensory network: decrease pre- to post-central gyrus connectivity.
Zhang *et al.* 2012 (see also 2015) [59,60]	Resting state fMRI	21 participants with PWS (11 males, mean 7.3 y) 18 sibling control participants (8 males, mean 11.1y)	ALFF increased in PWS in ACC, hypothalamus, & left amygdala and decreased in medial PFC and right amygdala. Granger causality analysis of direction of connectivity: Increased causal influence bilaterally from amygdala to the hypothalamus, from the ACC to the medial PFC right amygdala, from the medial PFC to bilateral amygdala, & from ACC to medial PFC in PWS compared to control participants. Altered directionality in effective connectivity in all pairwise analyses except the right amygdala to hypothalamus, although this increased in strength.

ACC: anterior cingulate cortex; ALFF: amplitude of low frequency fluctuation; AS: Angelman’s syndrome; BMI: body mass index; EEG: electroencephalography; ERP: event-related potential; fMRI: functional magnetic resonance imaging; ID: intellectual disability; m: months; OFC: orbitofrontal cortex; PET: positron emission tomography; PFC: prefrontal cortex; PWS: Prader-Willi syndrome; rCBF: regional cerebral blood flow; UPD: uniparental disomy; y: years.

**Table 3:** Studies reporting neurochemical investigations and neuroanatomy at the cellular level.

Study	Methods	Sample	Key findings
Akefeldt *et al.* 1998 [61]	Lumbar puncture: CSF analysed for metabolites of serotonin (5-HIAA), dopamine (HVA) & noradrenaline (HMPG)	13 participants with PWS (8 males, 0.4–23 y) 5 control groups: 15 typically-developing participants (11 males, 6 aged 3–8 y, 9 aged 18–25 y); 22 typically-developing children (16 males, 3–14 y); 8 children with autism (5 males, 3–13 y); 7 children with mixed neurological diagnoses (6 males, 0.3–15 y); 4 overweight children with ID but not PWS (0 males, 4–15 y)	Increased levels of 5-HIAA & HVA in PWS compared to all groups, most markedly for serotonin. Significant for 5-HIAA at corrected level compared to all control groups except the overweight and mixed neurological groups at the corrected significance level, but trend found. Significant for HVA at corrected level compared to typically-developing groups and all control participants combined. No association found between increased metabolites in PWS and age, BMI or severity of intellectual disability.
Ebert *et al.* 1997 [62]	Venepuncture: Plasma GABA levels	14 participants with PWS (6 males, 2–21 y) 9 children with AS (7 males, 2–17 y); 2 control groups: 7 moderately obese participants without ID (5 males, 2–20 y); 5 healthy weight children with ID (4 males, 3–17 y)	Mean GABA levels in plasmas significantly higher (2-3 times) in both PWS and AS groups than either control group, but no significant difference between PWS and AS groups.
Fronczek *et al.* 2005 [63]	Post-mortem: immunocytochemistry and image analysis system estimation of orexin neuron number in the lateral hypothalamus	7 hypothalami from individuals with PWS (3 males, 5 adults aged 25–64 y, 2 infants aged 6m & 3 y) 11 control hypothalami, matched for age, sex, post-mortem delay, fixation time and premorbid illness duration.	No difference in number of orexin neurons in hypothalamus found.
Goldstone *et al.* 2002 [64]	Post-mortem: immunocytochemistry & *in situ* hybridization to study NPY, AGRP, and NPY mRNA expression in the hypothalamus	6 obese adults with PWS (2 males, 25–64 y) 4 obese adults without PWS (2 males, 66–76 y) 22 control adults (13 males, 28–90 y)	Significant decrease of NPY and tendency to decreased NPY mRNA expression in all obese subjects, including PWS, but consistent with literature on inhibition on NPY in obesity. No significant difference in AGRP and NPY staining, or NPY mRNA expression, between PWS and obese adults without PWS. NPY/AGRP neurons show appropriate functioning.
Goldstone *et al.* 2003 [65]	Post-mortem: immunocytochemistry assessment of GHRH neuron number in infundibular nucleus/median eminence complex of the hypothalamus	6 adults with PWS (2 males, 25–64 y) 2 children with PWS (1 males, 0.5 & 0.75 y) 6 o6 control participants (13 males, 22 adults aged 28–90y, 4 children aged 0.4–0.75 y)	Higher GHRH neuron number in both control adults and adults with PWS who had prolonged premorbid illness, but no difference between PWS and control or obese adults without PWS.
Hayashi *et al.* 2011 [66]	Post-mortem: immunohistochemical analysis of GABAergic interneurons in superior frontal cortex & OFC, ACh neurons in the nucleus basalis of Meynert & PPN, & orexin-A and vasopressin in the hypothalamus.	6 month old female with PWS 3 control subjects following fatal pneumonia (2 males, 4 m, 1 y, 6 y)	GABAergic interneurons in cortex and ACh neurons in nucleus basalis similar in PWS and control samples. No clear abnormalities in orexin and vasopressin neurons in hypothalamus. Marked reduction of ACh neurons in PPN.
Lucignani *et al.* 2004 [67]	Resting state PET: ^11^C-flumazenil binding to evaluate GABA_A_ receptor functioning.	6 participants with PWS (2 males, 19.3–29.7 y, mean 24.6 y) 9 typically-developing participants (9 males, mean 25.9 y)	Significant binding reduction (7%) in cingulate. Reduced binding of *ca*. 3-6% in PWS in temporal and frontal cortices, and including insula (7%), but not significant when corrected for multiple comparisons. Trend to reduced binding in amygdala, caudate and thalamus (10-14%). Binding potential very similar in hippocampus, putamen and parietal and occipital cortices.
Martin *et al.* 1998 [68]	Lumbar puncture: CSF analysed for levels of oxytocin and vasopressin	5 participants with PWS (2 males, 16–21 y) 6 typically-developing participants (0 males, 21–28 y)	Oxytocin levels in CSF significantly higher in PWS, especially in females. Vasopressin levels significant lower in PWS females compared to control females, but not for males or combined sex groups.
Pasi *et al.* 1989 [69]	Post-mortem: immunoradiological assay of beta-endorphin levels in neural tissue	19 y old female with PWS	No clear beta-endorphin abnormality: rank of levels of beta-endorphin in areas of the brain on which there was prior reference literature (hypothalamus, medulla, periaqueductal grey, pons, & thalamus) was very similar, with the exception of the medulla.
Swaab *et al.* 1995 [70]	Post-mortem: thionine and immunocytochemical staining to assess PVN size and number of oxytocin and vasopressin neurons	5 adults with PWS (2 males, 22–64 y) 27 control adults (14 males)	PVN significantly smaller (28%) in PWS, with total cell number reduced by 38%. Oxytocin number significantly decreased in PWS (by 42%) as was volume of PVN containing oxytocin cells (by 54%). No significant difference in number vasopressin neurons between PWS and control samples.
Talebizadeh *et al.* 2005 [71]	Post-mortem: RT-PCR evaluation of gene expression of ghrelin, peptide YY and their receptors in the frontal, temporal, and visual cortices, pons, medulla and hypothalamus	3 individuals with PWS (0 male, 1 infant aged 1 y, 2 adults aged 32 y) 2 individuals with AS (4y old male, 43y old female) 6 control individuals (3 males, 1–72 y)	Expression detected in all brain areas in PWS, AS and control samples, with exception of PYY in pons for 1 PWS & 1 control subject.

ACh: acetycholine; AGRP: agouti-related peptide; AS: Angelman’s syndrome; BMI: body mass index; CSF: cerebrospinal fluid; EMO: early-onset morbid obesity; GABA: gamma-aminobutyric acid; GHRH: growth hormone-releasing hormone; ID: intellectual disability; HMPG: 4-hydroxy-3-methoxyphenyl-ethylene glycol; HVA: homovanillic; m: months; mRNA: messenger ribonucleic acid; NPY: neuropeptid Y; OFC: orbitofrontal cortex; PET: positron emission tomography; PFC: prefrontal cortex; PPN: pedunculopontine nucleus: PVN: paraventricular nucleus; PWS: Prader-Willi syndrome; RT-PCR: reverse transcription polymerase chain reaction; UPD: uniparental disomy; y: years; 5-HIAA: 5-hydroxyindoleacetic acid.

Van Nieuwpoort *et al.* (2011) reported reduced anterior pituitary size in 12 adults with PWS compared to a control group, but no significant difference in posterior pituitary size nor absence of the pituitary bright spot [35]. Conversely, Miller *et al.* (1996) found no significant difference in the height of the anterior pituitary gland in 15 children with PWS compared to either isolated growth hormone deficiency or typically-developing controls, although approximately 20% showed no posterior pituitary bright spot [26]. Further, although Miller *et al.* (2006) described a higher rate of pituitary abnormalities in PWS compared to a typically-developing sibling control group, a similarly elevated rate was seen in early-onset morbid obesity group, questioning the specificity of pituitary abnormalities to PWS beyond early obesity more generally [30].

A further sizeable proportion of original research studies reporting anatomical neural findings in PWS were studies primarily concerned with characterising epilepsy in PWS. The majority of these studies involved retrospective case reviews of individuals with PWS attending particular services to ascertain the rate of epilepsy among these samples. With the exception of Gilboa and Gross-Tsur [16], only individuals presenting with seizures were further evaluated in regards to any anatomical abnormalities [15,20,25,34,36,37], thus largely confounding any assessment of the relationship between PWS and any anatomical irregularities found and the potential relationship to epilepsy. All studies reported the majority of individuals as presenting within normal limits according to their imaging evaluations, but where abnormalities were cited the most common were ventriculomegaly, agenesis of the corpus collosum and more general cortical atrophy [16,34,36,37]. The relationship of these abnormalities to the presence of epilepsy *versus* PWS is unclear and the general exclusion of individuals with PWS who don’t have seizures presents a major confound. However, the reported areas and types of abnormality are not dissimilar to those described in the wider PWS literature.

Iughetti *et al.*, (2008) also reported wider abnormalities in 10 of the 91 children in their study, namely ventriculomegaly in eight and thin corpus callosum in two [12]. A case series reporting neural investigations in four children with PWS following critical illness (three post-mortem, one MRI) found no clear consistent abnormalities; an array of varying neuropathologies in both grey and white matter in the cerebral cortex were described in three, including gyrification and corpus collosum neuropathology, as well as midbrain, hindbrain and cerebellar abnormalities in one case, while the fourth was within normal limits [33]. Other reports include a six month old with mild ventriculomegaly, poor cortical myelination (including corpus callosum) and cerebellar abnormalities, a male two-year old showing third and fourth ventricle expansion and hypoplasia of the corpus callosum, another male child (2.6 year) with cerebellar and corpus callosum abnormalities, and a female infant showing abnormal cerebral gyrification, including an abnormal sylvian fissure revealing part of the insula [18,39]. Hashimoto *et al.* (1998) also reported mild ventriculomegaly in four of five children with PWS, frontal cortical atrophy in three, and myelination delay in one, but no abnormalities of basal ganglia, thalamus, white matter and cerebellum [17]. Proton magnetic resonance spectroscopy indicated reduced *N*-acetylaspartate in two children, with decreases in this marker of neuronal functioning indicating neuronal loss or dysfunction. No neuropathologies were found in the comparison group of 37 typically-developing children of the same age range. However, Mantoulan *et al.*, (2011) reported no clear structural abnormalities on the MRI scans of nine PWS teenagers (12.7 year-18.6 year) [24].

Considering the specificity of such abnormalities to PWS, Miller and colleagues published a series of analyses comparing participants with PWS to both typically-developing sibling controls and a group of individuals with early-onset morbid obesity. Miller *et al.*, (2006) reported white matter abnormalities in six of eight adults with PWS, varying in location and number but were most commonly in the orbitofrontal cortex [27]. However, none of the nine younger patients with PWS showed these lesions, nor did any of the typically-developing controls. Further, there were no significant differences between the presence, location or number of these neuropathologies between the PWS and early-onset obesity groups, with previous MRI scans for several patients in both groups indicating the later development of these abnormalities. Similarly, investigating cerebral and cerebellar volumes, Miller *et al.*, (2009) reported smaller cerebellar volume and decreased cerebellar/cerebral ratios in both PWS and early-onset morbid obesity groups compared to typically-developing siblings, but no significant differences between them [31]. No differences between the three groups were found in cerebral volume. As described above, similar rates of pituitary abnormalities were also found in PWS and early-onset morbid obesity groups [30]. Consequently, these abnormalities would appear common in PWS, although non-specific and not universal, with early obesity a possible factor contributing to their development.

Nevertheless, some such comparisons did suggest anomalies in the PWS brain not frequently seen in early onset morbid obesity. Miller *et al.*, (2007) reported ventriculomegaly in all of 20 PWS participants, including infants for whom this was frequently the only abnormality present [29]. All participants over five years of age showed reduced parietal-occipital grey matter volume. Sylvian fissure polymicrogyria was reported in 60% and incomplete insular closure in 65%. No association with BMI or behavioural problems was found for any of these abnormalities and, with the exception of unilateral sylvian fissure polymicrogyria in two sibling and two early-onset obesity controls, these abnormalities were only seen in the PWS group. Looking more specifically at Sylvian fissure morphology in PWS, Miller *et al.* (2007) described incomplete closure of the insula in PWS compared to both a typically-developing sibling and early-onset morbid obesity control groups, with no significant differences between the control groups [28]. The closure of the insula is part of the process of cortical gyrification as the cortex develops [72], suggesting aberrant cortical development. However, in a comparison of cortical morphology in four children with PWS and six children with AS, Leonard *et al.* (1993) assessed the Sylvian fissure for abnormalities, measuring its features on MRI and qualitatively categorising each case, finding very few abnormalities in PWS, with many more in AS [21].

Larger quantitative assessment of cortical development is needed, rather than manual measurement and categorisation, to assess potential aberrant cortical gyrification more fully. Lukoshe *et al.*, (2014) recently published one such study looking at gyrification in children with PWS [23]. Using quantitative MRI, Lukoshe and colleagues looked at the local gyrification index (the ratio between total brain surface area and the surface area of a local defined region) in children with PWS compared to age and sex matched siblings to assess cortical complexity in PWS, finding reduced cortical complexity in frontal, temporal and parietal lobes. In particular, there were differences between the local gyrification indices in four largely frontal clusters, including insula and cingulate areas, but also areas of the parietal and temporal lobes. Within the frontal clusters, there were moderate correlations of the local gyrification indices with IQ, although the strength and significance of such correlations differed across clusters and IQ scale (total, verbal and performance IQ). The authors note the previous reports of decreased gyrification with intellectual disability and suggest this indicates early deviations in brain development in PWS [73]. In a further analysis of this data, Lukoshe *et al.*, (2013) described significantly smaller brainstem volumes, and trends towards reduced white matter volume and total cortical surface area compared with the sibling control group [22].

A small number of other quantitative studies have also indicated abnormalities in both grey and white matter. Ogura *et al.*, (2011) used voxel-based morphometry (VBM) to analyse MRI data from 12 participants with PWS compared to 13 age and gender-matched healthy-weight control participants, reporting an overall reduction in total brain tissue volume, grey matter volume and white matter volume, with specific reductions in the orbitofrontal cortex (OFC) and somatomotor areas [32]. Yamada *et al.*, (2006) described abnormalities in frontal white matter, the posterior limb of the internal capsule and the splenium of the corpus callosum compared to age and gender-matched controls in a diffusion tensor imaging (DTI) study [38].

Differences between the genotypes have also been reported. Honea *et al.*, (2012) reported a VBM analysis indicating smaller grey matter volume in the prefrontal and temporal cortices and reduced white matter volume in the parietal cortex in the deletion subtype, while those with UPD had smaller grey and white matter volume in orbitofrontal and limbic cortices [19]. However, those in the UPD group were, on average younger than those in the deletion group (mean 17.4 year *vs.* 25.2 year), which may present a confound owing to maturation of the brain, particularly of frontal regions, during this period of young adulthood. Lukoshe *et al.*, (2013) reported increased ventriculomegaly in UPD compared to deletion as well as surface cerebrospinal fluid (CSF), suggesting increased atrophy or differential development in UPD in childhood [22]. Lukoshe *et al.*, [23] additionally reported that two of the clusters in their findings had smaller local gyrification index values in participants with UPD compared to deletion, and that those with UPD had greater cortical thickness in one of these more frontal clusters. Contrasting neural structure between the genotypes and the relationship of any differences to those reported in the behavioural phenotype is understudied, perhaps owing to difficulties in recruiting adequate samples of those with UPD.

### Discussion of Anatomical Findings

A considerable proportion of studies reporting anatomical abnormalities in the PWS brain were case studies or case series, including both neuroimaging and post-mortem evaluations. The single case qualitative nature of these reports clearly limits the generalisability of these findings, as does the presence of sudden death or critical illness in Stevenson *et al.*’s study [33]. The varying ages of the patients studied could be involved in the discrepancies described, owing to the different timings of maturational development across areas of the brain. Nevertheless, the case studies, despite their limited generalisability suggested abnormalities including ventriculomegaly, aberrant cortical gyrification and atrophy and corpus callosum and cerebellar abnormalities. Cerebellar abnormalities may be implicated in the hypotonia, while ventriculomegaly already present at such young ages may indicate abnormal development of the cerebral hemispheres and is often seen in genetic disorders associated with intellectual disability [29]. Reduced cortical gyrification has been also associated with cognitive difficulties and likely implicates atypical processes of neuronal development [73].

This is supported by studies of larger PWS samples, which have described diffuse areas of abnormality, including reports of possible reductions in total white matter and grey matter volume as well as total cortical surface area. More specifically, reported grey matter abnormalities include aberrant cortical gyrification largely of frontal cortex, but also extending to temporal and parietal areas, as well as reduced grey matter in the orbitofrontal cortex and somatosensory areas. White matter atypicalities have been observed across frontal areas, as well as the large fibre bundles of the corpus callosum and the internal capsule. However, findings across studies are not always consistent and these reports need replication. Potential reasons for conflicting findings warrant further investigation, including between subtypes, clinical features and age, with many studies using participants spanning the lifetime from infancy through to middle-age, as well as the differing methodologies employed by each study. Many studies, including those describing multiple patients involved retrospective evaluations relying on previously acquired neuroimaging data, for which the contrasts used may differ widely, potential affecting the interpretation of findings across cases. However, many of the more systematic and controlled studies have also highlighted similar areas of neuropathology, including those where the image analysis was more quantitatively assessed without subjective or qualitative interpretation or categorisation by the researcher.

The majority of these studies did not directly investigate the association of abnormalities with the phenotypic features of PWS. Nevertheless, most authors note the intuitive associations that can be drawn between the regions where abnormalities were noted and the common features of PWS. Brainstem abnormalities may be involved in the pain, sleep and respiration difficulties associated with PWS, cerebellar abnormalities may be implicated in the hypotonia, and cortical abnormalities, particularly of the frontal areas, may be involved in the intellectual disability and executive function deficits often seen in PWS. More direct assessment of potential associations between abnormalities of structure and the presenting PWS phenotype is warranted.

## 4. Functional Studies in the PWS Brain

### 4.1. Food-Related Function

The majority of studies considering function in the PWS brain have investigated the overeating behaviour. These have highlighted dysfunction in neural networks involved in satiety and reward. Hinton *et al.*, (2006) used positron emission tomography (PET) to investigate the neural response of 13 participants with PWS while viewing food images following an overnight fast, a 400 kcal meal and a 1200 kcal meal [43]. They observed a similar neural response to that seen in a previous study of healthy weight individuals without PWS when fasted, with activation of the hypothalamus, amygdala, basal ganglia, thalamus, anterior cingulate cortex (ACC) and lateral OFC. However, the neural response associated with satiety, including increased activity in the medial OFC, temporal cortex and prefrontal cortex (PFC) was not found, even after the 1200 kcal load. Importantly, Hinton *et al.*, [43] were also able to demonstrate that the neural representation of satiety was not entirely absent: only half the participants showed a substantial change in ratings of fullness following the 1200 kcal meal, but in those who did there was a shift in increased activity from the lateral to medial OFC. Hinton *et al.*, [43] suggested this shift may result from the reward system dissociations of these regions, with lateral OFC implicated in mediating punishment value and medial OFC in reward value, with the higher calorie meal leading to a more satisfied state for those reporting increased fullness.

Similarly, Shapira *et al.*, (2005) reported a significant delay after an oral glucose load in the neural response in brain areas implicated in satiety compared to a previous study with obese participants without PWS and lean participants, at a mean latency of 24 min for the PWS participants, but only 15 and 10 min for the obese and lean participants respectively [54]. This only remained statistically significant for the ventromedial PFC when corrected for multiple comparisons, although the sample size was small (three participants). Miller *et al.*, (2007) reported greater PFC activation in PWS compared to healthy control siblings during the postprandial phase following an oral glucose load, in particular significantly increased ventromedial PFC activity, when viewing pictures of food compared to pictures of animals or tools, suggesting this may represent an increased reward value of food in PWS [51].

A further analysis of Hinton and colleagues’ MRI data showed that the high or low incentive value of food items, as rated previously by the participants and stable over time, was not associated with altered activity in the amygdala or medial OFC [44]. These areas are typically involved in incentive motivation, suggesting that any incentive motivation typically driven by food preferences may be overridden by the lack of satiety. However, Dimitropoulos and Schultz (2008) observed a possible effect of preference, or at least calorie content, on food-related neural responses in PWS [41]. Following a fast of at least three hours, participants with PWS showed greater activity in the hypothalamus, amygdala, insula and OFC in response to highcalorie compared to low calorie images than a control group of mixed aetiology ID participants matched for IQ and BMI.

Holsen *et al.* (2006) also used neuroimaging (fMRI) to look at the neural response to food images in PWS in areas implicated in reward and motivation; finding reduced activity in bilateral medial PFC and OFC in PWS when viewing food images in a pre-meal condition compared to a healthy weight control group, but increased activity in bilateral medial PFC, OFC and amygdala after a 500kcal meal [45]. Holsen *et al.* (2012) further described increased activation of the amygdala, hypothalamus and hippocampus in the PWS group compared to both healthy weight and obese control groups after a 500kcal meal, while the obese group exhibited greater activity in areas implicated in inhibitory control, including the dorsolateral PFC and the OFC [47]. Consequently, again, this study suggests that a combination of hyperactivity in regions implicated in food motivation and reward circuitry alongside hypoactivity in areas involved in inhibitory control may be responsible for the hyperphagia seen in PWS.

Differences between the genetic subtypes in regards to the neural response concerning food have been described. Holsen *et al.* (2009) looking at the response to food images in nine deletion participants and nine UPD participants, found that the deletion group exhibited greater pre-meal activity in the medial PFC, insula and amygdala—areas implicated in reward and affective motivation [46]. Post-meal, the greater medial PFC activity exhibited by the deletion group persisted, suggesting continuing increased reward signalling when viewing food images. In contrast, the UPD group showed greater post-meal activity in the dorsolateral PFC and parahippocampal gyrus, suggesting the greater recruitment of higher order cognitive control networks in coordinating the post-meal neural response to food images. However, no differences between genetic subtypes were discernible using the Three Factor Eating Questionnaire (TFEQ [74]). Both groups showed increased activity both pre- and post-meal in areas implicated in food motivation compared to healthy weight controls.

Consequently, the majority of studies investigating food-related functioning in the PWS brain have looked at the neural response to food images under different conditions of prior food intake. Both PET and fMRI evaluations suggest similar dysfunction, with subcortical areas implicated in motivation to eat showing prolonged or increased responding, while the responses inhibitory structures of the cortex appear to be delayed.

Investigating a different aspect of food motivation processing, Key and Dykens (2008) used EEG to look at neural discrimination of food composition and quality (single, combined in a palatable or unpalatable way or contaminated) between UPD, deletion and age-matched control groups [48]. Looking at the earlier N1 response, associated with perceptual characterisation, Key and Dykens found that those with deletion tended to process stimuli by quantity and those with UPD by quality, with controls more similar to those with UPD. The later P3 response, thought to reflect more cognitive motivational processing, indicated that those with deletion discriminated between palatable and unpalatable combined food stimuli, with response to the palatable stimuli greater than both the UPD and control group. This effect was present in the UPD group, although less pronounced, however this later event-related potential (ERP) was not present in the control group. The authors suggested that the reduced food motivation in controls may have prevented further processing of the images. EEG has greater temporal sensitivity, allowing the assessment of these very acute processes in a way that is not possible with imaging modalities such as PET and MRI. However, the ability to spatially localise any abnormalities is greatly reduced. The increase P3 response reported here may be related to the sustained motivation towards food images reported in the MRI and PET studies described above, with both genotypes showing greater dysfunction compared to controls. It is worth noting that both this EEG study and Holsen *et al.*’s [46] fMRI study comparing PWS genetic subtypes suggest that the deletion subtype may show greater atypicality, with those with UPD more similar to controls, although still dysfunctional. Combining these modalities within similar tasks may enable greater deconstruction of aberrant neural processing in both location and time.

### 4.2. Function Relating to Other Aspects of the PWS Phenotype

Atypical frontoparietal activation during set-shifting tasks has been postulated to underpin the need for routine and, by extension, temper outbursts, with Woodcock and colleagues reporting that a higher score on a questionnaire measure of repetitive behaviours positively correlated with severity of attention switching impairment during a computerized set-shifting task, as well as increased outburst-related behaviour exhibited during set-shifting and in response to unexpected change during more naturalistic activities [75,76,77]. In a matched-performance, cued task switching paradigm with eight individuals with PWS and eight typically-developing age and gender matched control participants, Woodcock *et al.*, (2010) reported hypoactivity in frontoparietal regions, including the posterior parietal and ventromedial prefrontal cortices in the PWS group during switching [57]. The authors suggest this may relate to impairments in setting appropriate attentional weights as task rules shift. Woodcock *et al.* [57] also reported a group interaction effect with the hippocampus, amygdala, thalamus, and putamen exhibiting increased activation in the typically-developing group compared to the PWS group during switching. Activity in these areas did not differ significantly within the control group for switch compared to same task demands, with this difference arising due to the deactivation of these regions in the PWS group during switching. The PWS participants also failed to show the deactivation of areas in the bilateral frontal poles. Therefore, the authors suggest that, taken together, these findings may indicate a lack of activation of in the task-positive regions of the default mode network (DMN) and deactivation of task-negative areas, with dysfunction in this network previously associated with deficits in attentional control. Woodcock and colleagues propose that these difficulties with set-shifting underlie the behavioural problems experienced by many people with PWS. Consequently, the research group has been investigating the use of “change cards” to flag up and prepare individuals for a change, in order to make the change more explicit and less unpredictable, reporting some improvement in behavioural response [78]. This does suggest that it is specifically *unexpected* change that is difficult in PWS, with more time to process a change enabling the individual to better respond to it. However, the predictability of changes was not directly tested in the cognitive tasks of set-shifting, where blocks of one task were followed by another after instruction regarding the change and thus the change was highlighted and not strictly unpredictable. Nevertheless, impairment in task performance was still found. Manipulating the predictability of switching in task runs may provide greater insight into the nature of this cognitive impairment in PWS, including the extent and type of preparation for change that may be most effective for reducing difficult behavioural antecedents.

Other facets of cognition have also been considered in PWS. Using EEG, Stauder *et al.* (2002) looked at the P3 response to oddball tasks to investigate visual and auditory impairment in PWS [55]. The oddball usually evokes a positive P3 response that has maximal amplitude at central parietal electrode sites approximately 300 ms after the stimulus onset and is associated with memory processing and stimulus evaluation, with the amplitude particularly associated with depth of processing. Whilst the PWS group could perform the tasks, the P3 ERP amplitude was reduced compared to a healthy control group, with greater impairment in the auditory modality in PWS. The authors suggest that it may reflect short term memory deficits in PWS, based on previous findings of short term memory impairment in PWS and prior associations with the P3 ERP. Such impairment in working memory may not be independent from difficulties with set-shifting, which require stimuli to be monitored and different task sets to be kept online and reconfigured as appropriate. Deficits in working memory may impede the successful performance of such processes. Nevertheless, the poorer in the auditory modality suggests a particular impairment. Akefeldt *et al.*, (1997) also reported potential auditory impairment, reporting possible abnormalities in the auditory brainstem response ERPs within their wider investigation of speech and language characteristics in PWS [40].

Some differences in the neural profiles of the UPD and deletion subtypes have been described in relation to aspects of the wider phenotype. Coding or processing speed has also been reported to differ between genotypes, being slower in those with UPD [79]. Neurophysiological evidence of this greater impairment in UPD comes from a study of ERPs during a go-nogo task [56]. While only the control group showed the both the typical N200 and P300 task modulation, related to early modality-specific inhibition and later general inhibition respectively, only the UPD group showed impaired late general inhibition alongside significantly poorer performance. As coding speed is also reported to be slower in both schizophrenia and vulnerability to the disorder [80,81], it is possible that this may be involved in the higher propensity of those with the UPD subtype to psychotic illness.

The UPD subtype has also been associated with an increased prevalence of autism spectrum conditions and related behaviours (e.g.,[82]). Halit *et al.*, (2008) investigated face processing in PWS, using EEG during face recognition and eye direction tasks, predicting that those with UPD would show greater deficits on these tasks of social cognition [42]. Behavioural scores were fairly poor, but did not differ between genotypes; however, the effects of face orientation and gaze direction on the N170 amplitude did. Contrary to prediction, the N170 amplitude in the deletion, not UPD group, failed to differentiate upright from inverted faces and direct from averted gaze. This suggests impaired social cognition in both groups with PWS, however the prediction of greater severity of dysfunction in UPD was not borne out. 

Subsequent to the literature search, but identified according to the criteria and updates from the databases initially searched, Klabunde *et al.*, (2015) recently reported an fMRI study of skin picking in PWS [50]. The researchers compared the neural response during periods of picking to periods when no skin picking took place. Increased activity was observed in a number of areas during skin picking, including two clusters of activity; one in the right ACC and right middle frontal gyrus and the other encompassing the primary somatosensory cortex, supplementary motor area, left middle frontal gyrus and right insula. Activity in both the right insula and the left precentral gyrus correlated negatively with the number of current skin picking sites. Since these regions are implicated in the processes of interoception, movement, attention and somatosensation, and are also activated during pain processing and itching/scratching, the authors suggest that interoceptive dysfunction may lead to the skin picking behaviours in PWS, with the behaviour reinforced because it helps to maintain a balance in a system in which there is clear impairment (such as in the interoception of satiety and pain).

### 4.3. Resting State

Other researchers have looked at activity in the brain in PWS at rest, often correlating areas of aberrant activity with behavioural measures. Using PET, Mantoulan *et al.*, (2011) analysed the regional cerebral blood flow (rCBF) in the absence of any task, reporting significant hypoperfusion in the limbic, superior temporal and parietal lobes, most strongly in the anterior cingulate and superior temporal regions, but also in the right orbitofrontal and postcentral gyri [24]. Moreover, significant associations were found between these areas and scores on the Childhood Behaviour Checklist, indicating the dysfunction found may be related to the social, behavioural and psychiatric disturbances. However, while the age range of this group of PWS participants was smaller than in many studies (12.7–18.6 y) the healthy control group were generally older (mean 21.2 y), presenting a potential confound given the rapid neural development expected at this time.

Kim *et al.*, (2006) [49] studied regional glucose metabolism using fludeoxyglucose (18F) PET in children with PWS, including those at the transitional phase between failure to thrive and overeating, compared to typically-developing relatives, following a fast of at least four hours [49]. In those with PWS, Kim *et al.*, [49] reported decreased metabolism in the right superior temporal gyrus, left verebellar vermis, right OFC, right temporal pole and left uncus. Increased metabolism was observed in the bilateral middle frontal cortex, left superior frontal cortex and right inferior frontal cortex, as well as the ACC. The authors note that these regions have all been implicated either directly or indirectly with cognition relating to eating, taste and food reward, emotion, and obsessive-compulsive behaviours, but these behaviours and skills were not directly measured. This finding of increased metabolism in the ACC contrasts with the hypoperfusion, particularly of the ACC and superior temporal regions, reported by Mantoulan *et al.*, [24] who suggest that the use of sedation in the current study may have contributed to this discrepancy. Other possible factors include the difference in mean age of the children in each study (4.2 y in Kim *et al.* [49] *vs.* 16.4 y in Mantoulan *et al.* [24]), with Kim *et al.* [49], reporting the inclusion of children at the transitional eating phase, as well as the fact that participants in Kim *et al.*’s [49] study had fasted prior to the scan and thus may have been in a different motivational state to those in Mantoulan *et al.*’s [24] study.

Ogura *et al.*, [52] studied regional rCBF at rest using single proton emission computed tomography (SPECT) in the same 12 participants and age-matched controls who participated in their anatomical MRI study described above [32]. Reduced rCBF was reported bilaterally in the lingual gyri and cerebellum and in the right thalamus and left insula, whilst increased rCBF was found bilaterally in the angular gyri and inferior frontal gyri and in the left middle frontal gyrus. In this study, a questionnaire assessing eating, stereotyped and collecting/hoarding behaviour was administered, with the eating severity score correlating negatively with rCBF in the left insula—an area associated with conscious interoception of internal states. These participants with PWS were older than those in either Kim *et al.* [49] or Mantoulan *et al.*’s [24] studies, but closer in age to the latter and, similarly, remained unsedated during the scan. The findings bear some similarity to those of both prior studies, but appear to show greater consistency with those of Kim *et al.*, [49] suggesting neither sedation nor age may be the main explanatory variable. Perhaps crucially, the participants taking both Ogura *et al.* [52] and Kim *et al.*’s [49] study were fasted, whilst Mantoulan *et al.*, [24] do not report a fasted state in their protocol.

Considering how alterations in distinct neural areas may lead to network dysfunction, Zhang *et al.* (2013) studied functional connectivity in 21 children with PWS using resting-state fMRI in four resting state networks (RSNs) encompassing regions implicated in eating behaviour: the DMN, the core network, the prefrontal lobe network and the sensorimotor network [58]. The authors ascertained regions of interest (ROIs) by identifying regions which showed significant differences in activity at rest between the PWS participants and a healthy sibling control group. Increased amplitude of low frequency fluctuation (ALFF) was reported in the PWS group compared to the control group in the bilateral ventrolateral PFC, ACC, inferior parietal lobe and left insula, while decreased ALFF was found bilaterally in the medial and dorsolateral PFC, hippocampus, precuneus, and pre- and post-central gyri, and the leftOFC. This largely supports Kim *et al.*’s [49] findings of increased rCBF in the ACC, but contrasts with Ogura *et al.*’s [52] reports of decreased activity in the insula and both studies accounts of increased medial PFC activity. Again, the difference in conditions of fasting may be implicated.

The pairwise functional connectivity strengths between these regions were analysed within the four RSNs of interest. All networks showed alterations in functional connectivity. Within the DMN there was reduced functional connectivity strength between the inferior parietal lobe and the medial PFC, precuneus and hippocampus, as well as between the medial PFC and precuneus, echoing Woodcock *et al.*’s [57] findings of aberrant recruitment of frontoparietal areas during set-shifting. In the sensorimotor network decreased functional connectivity between the pre and post central gyri was reported. The prefrontal cortex network showed reduced connectivity strength between the dorsolateral PFC and OFC, but increased strength between the ventrolateral PFC and the OFC and dorsolateral PFC. Increased functional connectivity strength was also observed between the ACC and the insula in the core network. The authors related these differences to differences in reward and food regulation processing in PWS, with these areas frequently found to show aberrant activity in the studies in which participants viewed food images (see 4.1). However, no direct measures of eating behaviour in these participants were taken, nor were the findings considered in light of other PWS characteristics.

Zhang *et al.*, (2012) [59]; also published as a full length article subsequent to the literature search [60] took this analysis further, assessing the directionality of causal influence in functional connectivity using Granger causality analysis, using the medial PFC, ACC, amygdala, and hypothalamus as ROIs based on their previous implication in the aberrant eating behaviour in PWS . They reported increased ALFF in the ACC, hypothalamus and left amygdala, with decreased ALFF in the medial PFC and right amygdala; again, these are areas typically highlighted by studies using a food image viewing task and strongly implicated in food-related motivation. Zhang and colleagues reported increased Granger causality bilaterally from the amygdala to the hypothalamus, from the ACC to right amygdala, from medial PFC to bilateral amygdala, and from ACC to medial PFC in PWS. Furthermore, altered directionality in effective connectivity was found between all pairwise combinations of these ROIs, with the exception of the right amygdala to the hypothalamus in which directionality was the same as the control group but markedly increased in strength. The authors argue that this shows abnormal connectivity and strength of the “driving force” in food-related circuitry at rest in PWS, which appears to involve widespread atypical functioning across a distributed network. Although not considered in the study, these areas are also implicated in emotional cognition and regulation and may also indicate aberrant network activity involved in other features of PWS, such as mood lability and related maladaptive behaviours.

Resting state neuroimaging presents advantages, particularly in patient populations who may not be able to adequately complete tasks or whose performance may differ so greatly to controls as to be incomparable, however it does not come without its limitations. In particular, functional connectivity has been criticised for susceptibilty to motion artefacts. Pujol *et al.*, (2014) studied the effects of motion in resting state fMRI in children, young adults, older adults, PWS, Down’s syndrome and William’s syndrome [53]. In young adults (the group who moved least), motion correlated with connectivity in a system-specific way, showing positive correlations in sensorimotor cortex and visual areas but negatively with areas of the DMN, likely reflecting neural function related to the motion rather than actefactual error. Different regions were found to correlate with motion in each developmental disorder, with motion in PWS mainly correlated with frontal and temporal basal regions, including the hypothalamus, but also with the dorsal ACC. The authors assert the need to consider carefully not only motion effects on connectivity but also the effects of correcting for them, particularly as these may differ according to extent of motion and disorder and may be distinct according to disorder.

### 4.4. Discussion of Functional Findings

Research to date is weighted towards exploration of the prominent hyperphagia seen in PWS, with studies describing a significantly delayed satiety response, with aberrant activity in areas associated with reward and motivation and many of the resting state findings interpreted in light of the eating behaviour only. In comparison to food-related neural function, mechanisms underlying the further cognitive, behavioural and psychiatric features have been less researched and are currently poorly understood.

Briefly, hyperactivity of regions associated with reward or motivation, such as the hypothalamus and amygdala, and hypoactivity of regions implicated in inhibitory control (typically frontal cortex) are typically observed during food-related tasks. Hypoactivity was also described in Woodcock *et al.*’s [57] work investigating attention switching in PWS, with frontoparietal areas failing to be adequately recruited, and increased activity in areas of the DMN, which includes the hippocampus, medial PFC and other areas which remained hyperactive in the food-based functional reports. Studies of the brain at rest in PWS have reported aberrant activity most notably in similar areas to those implicated in the task-based studies, including the ACC, medial PFC, OFC, dorsolateral PFC, thalamus, insula, hippocampus and hypothalamus, as well as in regions of the parietal and temporal lobes. This suggests widespread dysfunction across distributed neural circuits, supported by Zhang *et al.*’s [58,59,60] findings of altered connectivity strength between these areas, including between subcortical and cortical areas, which may be involved in the balance of subcortical motivational drive states and cortical integration of such information.

## 5. Neurochemical Investigations and Neuroanatomy at the Cellular Level

Other studies have focused on potential neurochemical abnormalities in PWS. Swaab *et al.*, (1995) reported a decrease in the size of the paraventricular nuclei (PVN) of the hypothalamus in five individuals with PWS compared to 27 control PVN samples [70]. This reduction corresponded to a significantly and marked reduction in the number of oxytocin-expressing neurons in PWS as well as the volume of the PVN containing them, but no significant difference in the number of vasopressin cells. Oxytocin is known to play a role in inhibition of eating behaviour as well as social behaviour and the reduction anxiety and related arousal [83,84], and thus may explain to some extent the combination of behaviours in PWS. Conversely, however, Martin *et al.* (1998) reported increased levels of oxytocin in the CSF of five individuals with PWS compared to six control participants, especially in females [68]. Vasopressin levels did not differ significantly between the PWS and control groups, except when split by gender, when vasopressin levels were significantly reduced in PWS females compared to control females. The PWS participants were younger (16–21 y) than the control participants (21–28 y), which presents a potential confound. Nevertheless, both studies suggest dysfunction of the oxytocin system in PWS, and it is important to note the ability for both upregulation and downregulation of activity in impaired systems.

Dysfunction of neurochemical systems in PWS is also unlikely to be specific to the oxytocin system with Akefeldt *et al.* (1998) reporting increased serotonin and dopamine metabolites in the CSF in PWS compared to a number of healthy and disorder control groups combined, most significantly for serotonin [61]. When compared separately to the overweight group and mixed intellectual disability groups this was not significant, and may indicate a role for weight and ID rather than specifically the presence of PWS. However, no association between the metabolite levels and BMI or IQ was found, and a significant difference was seen between the PWS group and a group with autism including low IQ. Moreover, the overweight group, in particular, was very small, meaning there may have been little power to detect a significant difference; a trend in the right direction was found.

Lucignani *et al.*, (2004) investigated gamma-aminobutyric acid (GABA) A receptor function in six young adults PWS compared to typically-developing control participants using resting-state positron emission tomography of the benzodiazepine binding site (carbon-11 flumazenil binding), since the genes encoding three GABA_A_ receptor subunits are found within the PWS critical region [67]. ROI analyses found reduced binding in frontal and temporal cortices, notably in the insula and cingulate cortex, although only the cingulate was significant after correction for multiple comparisons. Trends towards reduced binding were also seen in the amygdala, caudate nucleus and thalamus, while binding in the parietal and occipital cortices, hippocampus, and putamen were similar to that in typically-developing controls. This suggests aberrant GABAergic function in PWS in both frontal and limbic cortical areas, as well as subcortical regions. Ebert *et al.*, (1997) also reported evidence for GABAergic dysfunction using plasma GABA levels as a proxy for CNS levels, reporting plasma GABA levels 2-3 times higher in both PWS and AS compared to both obese controls without ID and healthy weight controls with ID of mixed aetiology [62]. A better understanding of the relation of this finding to reduced binding in Lucignani *et al.*’s [67] study would require further measurement of GABA levels within the central nervous system.

Single case post-mortem studies have also reported potential neurochemical dysfunction. Hayashi *et al.* (2011) reported a reduced number of acetylcholine neurons in a six-month old female compared to three controls in the pedunculopontine nucleus, with no significant difference in the number of acetylcholinergic neurons in the basal nucleus of Meynert, GABAergic interneurons in the cerebral cortex, nor vasopressin and orexin-A neurons in the hypothalamus [66]. The authors suggested a potential role of the acetylcholine neurons of the pedunculopontine nucleus in the sleep dysregulation and hypotonia seen in PWS, while the findings regarding vasopressin support the work of Swaab *et al.* [70]. The lack of GABAergic dysfunction reported here may result from the differering aspects of the GABAergic system studied (e.g., interneurons in this case in contrast to receptors or plasma levels). However, other authors have failed to find abnormalities, despite intuitive associations between particular neurochemicals and features of PWS. In line with Hayashi *et al.*’s [66] results, an earlier study by Fronczek *et al.*, (2005) also observed no significant difference in orexin neurons in the hypothalami of seven PWS patients compared to well-matched control samples (matched for age, sex, post-mortem delay, fixation time and premorbid illness duration), suggesting orexin is not directly implicated in the hypersomnolence seen in PWS [63]. Goldstone *et al.*, (2002) reported similar NPY and AGRP staining and NPY mRNA expression in the post-mortem hypothalami of six obese PWS patients compared to obese controls, although NPY and NPY mRNA expression was significantly reduced compared to healthy weight controls compared to all obese subjects [64]. This was consistent with the literature about effects of obesity on these peptides and was not present in two infants with PWS prior to hyperphagia and obesity onset (unpublished data reported in [64]), suggesting that NPY/AGRP neurons function appropriately in PWS, and any deviance from normative levels can be accounted for by factors such as obesity. In another post-mortem study, Goldstone *et al.*, (2003) also found no evidence for abnormal growth-hormone-releasing hormone neuron numbers in PWS, suggesting a reduction in neurons is not the primary cause of the growth hormone deficiency [65].

Talebizadeh *et al.*, (2005) looked at the expression of ghrelin and peptide YY and their receptors in post-mortem samples of six brain regions (frontal, temporal and visual cortices, and pons, medulla and hypothalamus) in three PWS patients and six control samples finding qualitatively similar expression in all samples, although a quantitative (and preliminary) analysis of expression levels was only carried out for the 1 y old PWS and control samples suggesting potential quantitative growth hormone receptor difference [71]. Finally, Pasi *et al.*, (1989) [69] reported no clear alterations of beta-endorphin levels pertaining to PWS in a single case post-mortem study of a young female adult with PWS, despite literature suggesting an association between beta-endorphin increases in CSF and appetite, pain insensitivity and thermoregulation difficulties [69].

### Discussion of Neurochemical Findings

Dysfunction in PWS appears unlikely to be specific to a singular neurochemical system, with abnormalities in a variety of diffuse neurochemical systems described to date. Some researchers have conducted anatomical investigations of cellular number, while others have looked at levels of neuropeptides or their metabolites. Replication of findings is clearly required, with a relatively small number of studies largely focusing on differing systems, and seemingly conflicting findings when the same system has been studied by different methods, such as post-mortem *vs. in vivo*, or when different aspects of the same system have been considered. However, dysfunction across the monaminergic, cholinergic and oxytocin systems in PWS seems probable. Further investigation of GABA dysfunction may be especially warranted, given the observations detailed above and the location of GABA receptor genes within the PWS region, although these are not thought to be imprinted. GABAergic dysfunction is believed to play an important role in psychiatric disorders, including psychosis [85,86], and may be involved in the high propensity to psychiatric illness seen in PWS.

## 6. General Discussion

Given the range of disturbances exhibited in the PWS phenotype, it seems likely that PWS involves aberrant activity across distributed neural networks and diffuse neurochemical systems. Research to date suggests that a combination of subcortical and higher cortical structures are involved, including those involved in processing reward, motivation, affect and higher order cognitive functions, with both anatomical and functional investigations indicating abnormalities. However, at present, characterisation of neural structure and function in PWS warrants replication and further systematic study. Given reports of atypical cortical gyrification, it is plausible that aberrant processes begin very early, possibly during the period of neuronal differentiation and migration. Preliminary evidence of differences in neuropathology and function between the two major PWS genetic subtypes is also available, which may be involved in corresponding variations in the expressed phenotype. Consequently, careful evaluation of connectivity in the PWS brain may prove fruitful in gaining inside into the pathway from PWS genetics to cognition and behaviour. In doing so, drawing on knowledge of networks and neural regions implicated in particular functions, including cognitive control, motivation, reward and affect, for example, will be essential.

In particular, the limbic cortico-striatal-pallido-thalamic loops, involving the orbitomedial prefrontal cortex, anterior cingulate cortex, ventral striatum, ventral pallidum and thalamus, with inputs from the hippocampus and amygdala appear to be implicated in emotional cognition, regulation and lability, modulated by the diffuse neurochemical systems [87,88]. A further potentially relevant network is the dorsolateral prefrontal network which has been strongly associated with executive functions, including the set-shifting ability reported to be impaired in PWS. The dorsolateral prefrontal network has connections with premotor and parietal regions and projects to caudate head, pallidum and thalamus, with inputs from premotor and parietal regions in the case of the former [87,88], with frontopariental activity reported to be aberrant during set-shifting in PWS [57]. 

In PWS, it appears likely that the difficulties with emotional regulation and the outburst behaviours may be associated with abnormalities in this circuitry. Many of these are areas which do appear to have been most consistently implicated in the literature regarding neural structure and function in PWS, whether anatomical, function during tasks or at rest. They have also been implicated to varying extents in the aetiology of disorders with which PWS shares some features, including depression and mood control, anxiety, obsessive-compulsive disorder, impulsive aggression (as differing from premeditated violence), and deficits in executive function [87,88,89,90,91,92,93,94,95].

Damage to the orbitomedial PFC has long been associated with deficits in emotional and impulse processing and regulation and use of emotional information in decision; for example, Damasio and colleagues’ findings that lesions to this area led to an absence of the automatic visceral response to emotive stimuli alongside an inability to take into account long-term consequences of actions (e.g., [96]). The interplay between central and autonomic nervous systems in regulating coordinated behavioural responses is considered in Porges’ polyvagal theory [97], which sees alteration of vagal tone as crucial in coordinating and integrating physiological and neural processes to form appropriate behavioural responses from the basic flight/flight reactions to more nuanced socially aware interactions. Importantly, this theory proposes that flexibility of behavioural responses crucially involves heart rate variability. Associations of activity in ventromedial PFC, an area repeatedly highlighted in this article, and cardiac function, via efferent vagal projections, have been reported [98]. Our group recently described preliminary reports of improvement of PWS-associated behaviours, with increased behavioural flexibility in an open trial of vagus nerve stimulation [99], potentially implicating this complex interplay in the maladaptive behaviours seen in PWS, with VNS demonstrated to influence activity throughout the brain, including the hypothalamus, OFC, amygdala, hippocampus, insula, medial PFC and cingulate cortex [89,100].

Consideration of neural networks in other disorders which appear to share some of the behavioural features associated with PWS and have already undergone more systematic neural investigation may provide a framework for investigation in a similar way to the application of research on food motivation and eating behaviour in healthy populations to the PWS population.

## 7. Conclusions

Much of the research on neural structure and function in PWS concern the prominent overeating behaviour seen in the disorder, with other features of PWS much less well defined at this level, often with only a singular study per feature. Anatomical abnormalities have been more extensively considered, although only more recently using the more quantitative techniques which have become available in neuroimaging with technological advances. The anatomy and function of the brain in PWS has also rarely been studied within the same samples. Nevertheless, across methodologies and designs, functional and anatomical studies have implicated a combination of subcortical and higher order structures in PWS, including those involved in processing reward, motivation, affect and higher order cognitive functions, potentially influenced by abnormalities in diffuse neurochemical systems. Further consideration of the specificity of abnormalities to PWS and their relation to the features and behaviours associated with the syndrome will be crucial in increasing our understanding of PWS and the development of therapeutic strategies.
